# Editorial: Rising stars in plant ROS/redox biology under abiotic stress conditions

**DOI:** 10.3389/fpls.2023.1207275

**Published:** 2023-05-26

**Authors:** Amith R. Devireddy, Rosa M. Rivero, Sara I. Zandalinas

**Affiliations:** ^1^ Biosciences Division, Oak Ridge National Laboratory, Oak Ridge, TN, United States; ^2^ Center for Bioenergy Innovation, Oak Ridge National Laboratory, Oak Ridge, TN, United States; ^3^ Department of Plant Nutrition, Centro de Edafología y Biología Aplicada del Segura - Consejo Superior de Investigaciones Científicas (CEBAS-CSIC), Campus Universitario de Espinardo, Murcia, Spain; ^4^ Department of Biology, Biochemistry and Environmental Sciences, University Jaume I, Castellon, Spain

**Keywords:** reactive oxygen species, abiotic stress, plant tolerance, nitric oxide, antioxidants, protein carbonylation, climate change

Plants are constantly subjected to a wide range of abiotic stresses including salinity, high temperatures, drought, or cold, among others, that negatively impact their growth and development and cause crop yield losses worldwide (Lesk et al., 2016; Bailey-Serres et al., 2019). These stress conditions induce the accumulation of reactive oxygen species (ROS) such as hydrogen peroxide (H_2_O_2_), superoxide ( $O_{2}.^{-}$ ), singlet oxygen (^1^O_2_), or hydroxyl radical (HO·). ROS play a key role in stress sensing and signaling, as well as in the activation of different mechanism involved in plant acclimation to different stresses (Mittler et al., 2022). However, ROS levels that are too high are cytotoxic (Mittler, 2017). To prevent cellular oxidation by ROS, an interplay between ROS production, scavenging and transport maintains ROS at controlled concentrations, as well as drives ROS signaling responses involved in plant acclimation to stress (Baxter et al., 2014; Mittler et al., 2022).

This Research Topic contains five original articles and one review article showing novel insights from recognized early career researchers (Rising Star researchers) into how ROS scavenging and signaling contribute to plant tolerance to different abiotic conditions. In particular, three articles report how changes in ROS metabolism, especially those associated with increased ROS scavenging, are involved in enhanced tolerance of tomato plants (Yang et al.) and fenugreek (Mohamadi Esboei et al.) to salinity. Additionally, methyl viologen (MV)-induced oxidative stress in Arabidopsis plants is subject of study (Melicher et al.). In particular, two manuscripts go in-depth in the study of the role of trehalose (Yang et al.) and melatonin (Mohamadi Esboei et al.) in the activation of antioxidants to protect tomato and fenugreek plants, respectively, against the damaging effects of salt stress. Foliar treatment of trehalose positively regulated photosynthetic parameters, osmolyte synthesis, and ion contents, and resulted in a higher antioxidant enzyme expression and activity, correlated with a decrease in ROS levels compared to untreated tomato plants under salt stress (Yang et al.). In turn, exogenous application of melatonin could be involved in enhancing the content of chlorophylls and carotenoids, as well as the biosynthesis of antioxidants and nitric oxide (NO), resulting in an enhanced protection of fenugreek from salt stress (Mohamadi Esboei et al.). In addition, the manuscript provided by Melicher et al. explored the antioxidant role of iron superoxide dismutase 1 (FSD1) in Arabidopsis under oxidative stress induced by MV at low Cu^2+^ concentrations. Briefly, FSD1 was positively correlated with APX function and PSII repair cycle, and prevented cytoplasmic degradation during oxidative stress at limited Cu^2+^ contents, suggesting that FSD1 plays a protective role during oxidative stress in a Cu^2+^-dependent manner (Melicher et al.).

Besides the importance of ROS scavenging for tolerance to abiotic conditions, ROS signaling is also known to play a key role in plant acclimation to stress. In this sense, the article by Terrón-Camero et al. discussed the role of redox-sensitive proteins in activating signal transduction events in plants. In particular, the article focused on transcriptomic studies related to altered ROS metabolism in plant peroxisomes and identified common transcriptional footprints for plant peroxisomal-dependent signaling of plants. The study highlighted that peroxisomal-dependent genes highly overlapped with genes responsive to different abiotic stresses such as paraquat, salt and heat shock stress, providing therefore a valuable resource for assessing key peroxisomal functions in cellular metabolism under control and stress conditions across species. In line with this context, the research article of Fangue-Yapseu et al. emphasized the role of carbonylated protein molecules in ROS signal transduction. With the aim to identify the carbonylated proteins involved in signaling pathways, the authors determined proteins responsive to carbonylation by exogenous application of H_2_O_2_. Results indicated a unique set of carbonylated proteins related to sulfate adenylyl transferases and amidophosphoribosyl transferases and highlighted the importance of protein carbonylation in H_2_O_2_ signaling and hormesis effects. In addition to ROS, NO can function as a signaling molecule in plant cells. In this context, the review article provided by Kabange et al., discussed the current knowledge of NO signaling and its interaction with other signaling pathways in plants. Specifically, the authors focused on calcium and hormonal signaling events under elevated greenhouse gases (GHGs) and signaling mechanisms underlying GHGs-induced stress in plants. In this regard, the authors discussed signaling aspects of elevated carbon dioxide or ozone-induced NO regulating plant physiological and biochemical changes, highlighting the changes in the antioxidant systems. To support their findings, the authors proposed various signaling pathway models involving NO in plants exposed to elevated GHGs. Lastly, the authors suggested that, concerning climate change, depicting the molecular basis of NO-mediated plant responses under elevated GHGs would help elucidate the regulatory mechanisms underlying plant response to these conditions.

In summary, the different reports contained in this exciting Research Topic support the already established importance of ROS scavenging and signaling pathways in plant tolerance to different abiotic stresses, highlighting the role of ROS, NO and protein carbonylation in plant physiology and response to environmental constraints. Therefore, these studies contribute to a better understanding of the mechanisms underlying plant stress response and may have implications for plant breeding and crop improvement.

## Author contributions

All authors listed have made a substantial, direct, and intellectual contribution to the work and approved it for publication.

## References

[B1] Bailey-Serres J. Parker J. E. Ainsworth E. A. Oldroyd G. E. D. Schroeder J. I. (2019). Genetic strategies for improving crop yields. Nature 575, 109–118. doi: 10.1038/s41586-019-1679-0 31695205 PMC7024682

[B2] Baxter A. Mittler R. Suzuki N. (2014). ROS as key players in plant stress signalling. J. Exp. Bot. 65, 1229–1240. doi: 10.1093/jxb/ert375 24253197

[B3] Lesk C. Rowhani P. Ramankutty N. (2016). Influence of extreme weather disasters on global crop production. Nature 529, 84–87. doi: 10.1038/nature16467 26738594

[B4] Mittler R. (2017). ROS are good. Trends Plant Sci. 22, 11–19. doi: 10.1016/J.TPLANTS.2016.08.002 27666517

[B5] Mittler R. Zandalinas S. I. Fichman Y. Van Breusegem F. (2022). Reactive oxygen species signalling in plant stress responses. Nat. Rev. Mol. Cell Biol. 23, 663–679. doi: 10.1038/S41580-022-00499-2 35760900

